# No Identifiable Primary, Only Metastases: Case Report of a Small Round Blue Cell Tumor Consistent With Ewing Sarcoma Presenting With Multisystem Spread in a Young Woman

**DOI:** 10.1177/11795476261457179

**Published:** 2026-06-01

**Authors:** Simra Irfan, Ahmed Muneeb, Mallick Muhammad Zohaib Uddin, Muhammad Ahmed, Saba Akram, Abdullah Muhammad, Wasim Memon, Uffan Zafar

**Affiliations:** 1 Department of Radiology, 66705 Aga Khan University Hospital Karachi, Karachi, Pakistan; 2 Medical College, 66705 Aga Khan University Hospital Karachi, Karachi, Pakistan; 3 Department of Pathology, 43989 George Washington School of Medicine and Health Sciences, Washington, WA, USA; 4 Department of Radiology, 12338 University of Texas Medical Branch, Galveston, TX, USA

**Keywords:** Ewing sarcoma, bone cancer, metastasis, low - and middle - income countries

## Abstract

Ewing sarcoma is the second most common malignant bone tumor in children and adolescents but remains rare and highly aggressive. We present the case of a 31-year-old woman with widespread metastatic disease suggestive of Ewing sarcoma, including lung, spinal, and brain involvement at presentation; however, no dominant primary lesion was identified despite extensive imaging. The patient’s clinical course was complicated by neuropathic pain, seizures, and severe treatment-related infections. She was managed with multi-agent chemotherapy and supportive care but ultimately left against medical advice due to socioeconomic constraints. This case illustrates the diagnostic and therapeutic challenges of Ewing sarcoma in low-resource settings and underscores the importance of early detection, molecular confirmation, and multidisciplinary management.

## Introduction

Ewing sarcoma (ES) is a rare yet highly aggressive malignancy primarily originating in the osseous tissues. It is recognized as the second most prevalent primary malignant bone neoplasm in the pediatric and young adult population, following osteosarcoma, contributing to approximately 6–10% of all childhood bone cancers.^1,2^ The defining molecular signature of this tumor is a chromosomal rearrangement, most commonly t.^3,4^ (q24;q12), resulting in the formation of the EWSR1–FLI1 fusion transcript.^1,5^ However, in resource-constrained settings, confirmatory molecular testing is often unavailable, leaving the diagnosis heavily reliant on histopathology and immunohistochemistry.

The disease primarily manifests within the diaphyseal regions of long bones or components of the axial skeleton, but it can also arise from extraosseous soft tissues.^2,6^ Approximately 20–30% of patients exhibit metastatic dissemination at the time of initial diagnosis, predominantly involving the lungs, skeletal system, and bone marrow.^2,6^ In advanced presentations with multifocal skeletal involvement, distinguishing a primary tumor from metastatic disease can be challenging. The designation of an ‘occult primary’ remains controversial when multiple osseous lesions are present,^3,7^ making it difficult to definitively isolate an obvious primary site without advanced metabolic imaging, such as positron emission tomography-computed tomography (PET-CT).

Therapeutic strategies for ES are inherently multimodal.^2,6,8^ Our clinical case of a 31-year-old female with widely disseminated disease, suggestive of Ewing sarcoma, highlights the aggressive natural history of the malignancy, particularly when central nervous system involvement is present at diagnosis. Strikingly, her clinical course was further complicated by refractory neuropathic pain, breakthrough seizures, and severe treatment-related complications, reflecting the multifactorial challenges of delivering optimal oncologic care in a resource-constrained setting.^9,10^

## Case Report

**Figure 1.**
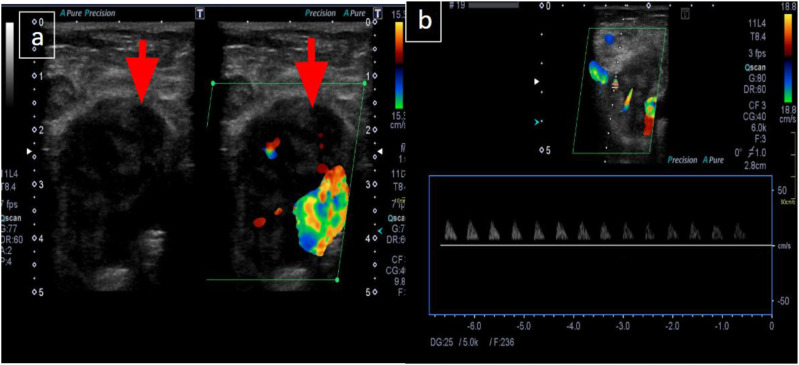
(A and B). Pelvic ultrasound demonstrating a vascular, lobulated hypoechoic lesion in the left inguinal region

A woman in her early 30s with no prior comorbidities presented with a two-month history of progressive left leg pain and palpable swelling, associated with low-grade intermittent fever and unintentional weight loss. Over the preceding week, she had developed generalized weakness along with urinary and fecal incontinence. On admission, her hemoglobin was 4 g/dL, for which she received three units of packed red blood cells. An X-ray of the left foot revealed a pathological talus fracture. An ultrasound of the left leg, initially performed to rule out deep vein thrombosis, was negative for DVT but incidentally identified large, heterogeneous, vascular structures in the left inguinal region and popliteal fossa^11^ (Figure 1).

**Figure 2.**
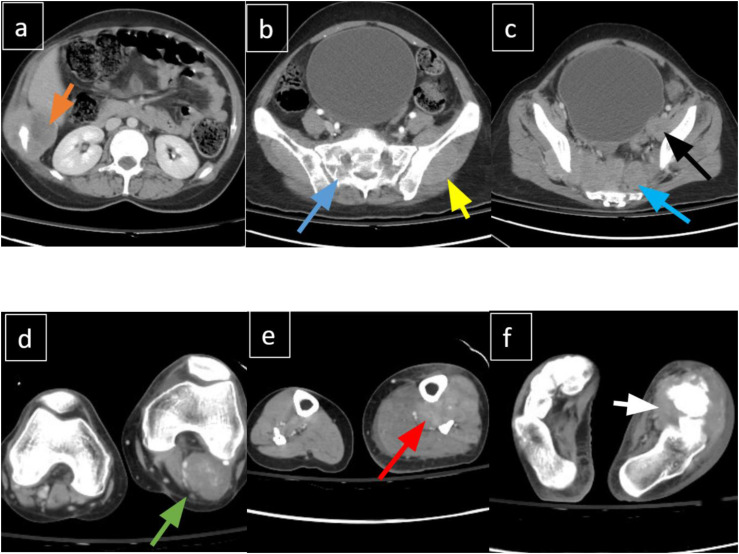
(A-F). Contrast-enhanced CT demonstrating widespread metastatic disease. (A) Right rib lesion with soft tissue component. (B and C) Pre-sacral mass with sacral infiltration, left iliac lesion, and local lymphadenopathy. (D-F) Enhancing soft tissue masses in the left popliteal fossa, lateral leg, and foot

A subsequent CT angiogram incidentally demonstrated widespread soft tissue lesions and extensive lymphadenopathy in the abdomen, pelvis, and thorax, along with lytic lesions in the vertebrae, sacrum, and right iliac bone (Figure 2). Additional findings included multiple pulmonary nodules in the left lung, compression of the left common femoral vein, and soft tissue edema of the left leg.

**Figure 3.**
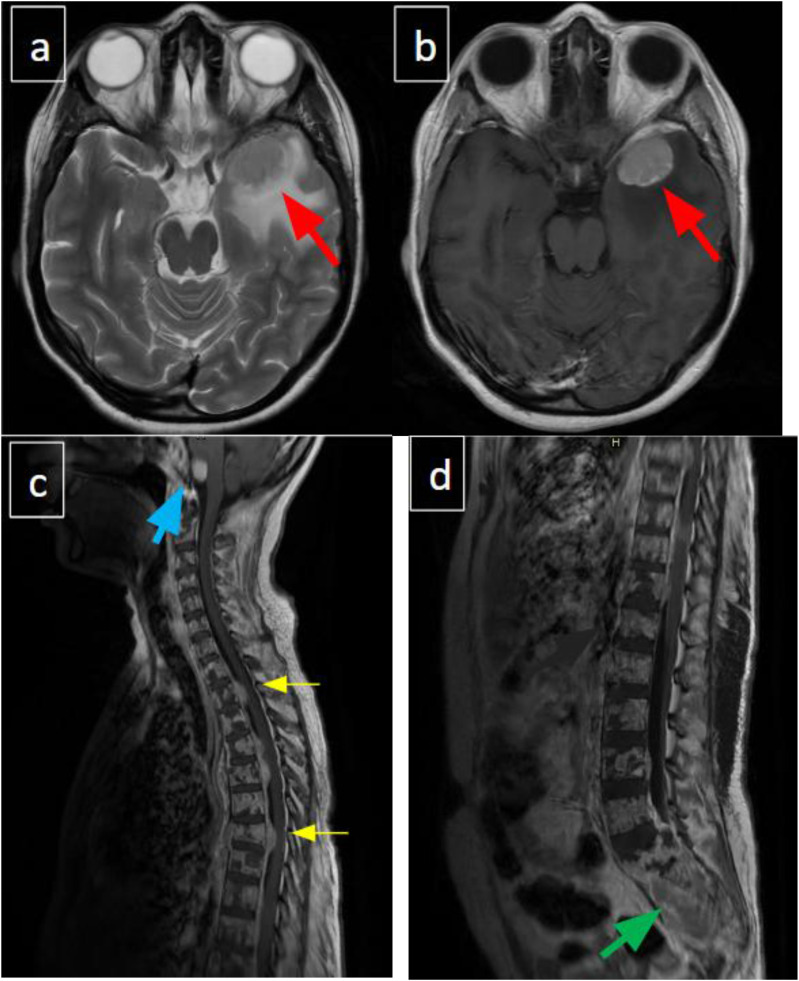
(A-D). Contrast-enhanced MRI. (A and B) Dural-based enhancing metastasis in the left middle cranial fossa with temporal edema. (C and D) Diffuse vertebral metastatic infiltration and large sacral lesion

MRI of the brain and whole spine with contrast exhibited severe metastatic disease involving both the brain and the vertebral column (Figure 3). A CT scan of the chest confirmed multiple bilateral pulmonary nodular infiltrates. Due to financial constraints, a PET-CT scan could not be performed to comprehensively map metabolic activity and definitively isolate a clear primary site among the widespread skeletal and soft tissue lesions.

**Figure 4.**
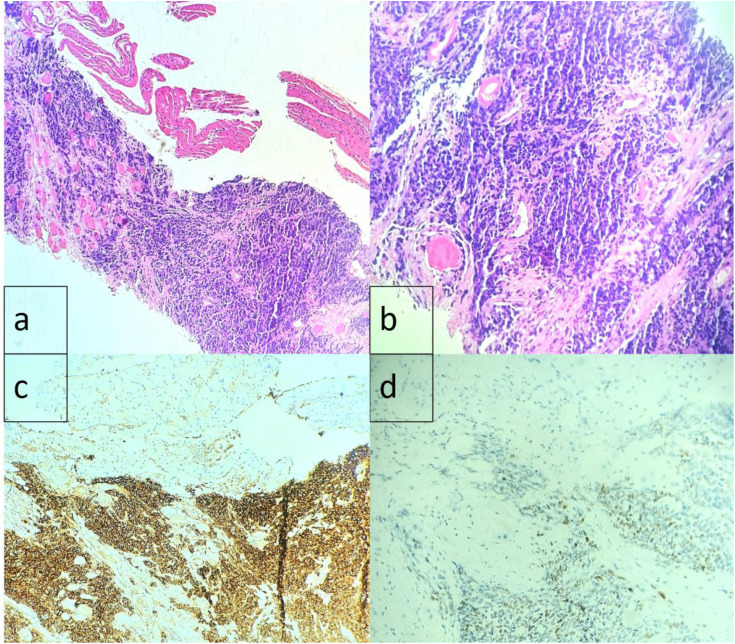
(A-D). Histopathology and immunohistochemistry. (A and B) Small, round, blue tumor cells infiltrating muscle fibers (H&E). (C) Diffuse membranous CD99 positivity. (D) Patchy NKX2.2 positivity

An ultrasound-guided biopsy of the presacral mass revealed histopathological features consistent with a small round blue cell tumor, favoring Ewing sarcoma. Histopathologic examination showed cores of fibro-muscular tissue infiltrated by a neoplastic lesion arranged in sheets (Figure 4). The tumor was composed of sheets of small, round, blue cells with scant cytoplasm, round nuclei, and dispersed chromatin.^7,12^ Immunohistochemically, there was strong diffuse membranous positivity for CD99 (MIC2 antigen).^7,12^ NKX2.2 showed patchy positivity, and Cyclin D1 was positive, while other markers (Cytokeratin AE1/AE3, Desmin, CD20, CD3, TdT, PAX5) were negative. The Ki-67 index was raised, indicating high proliferative activity.^2,7^ Although these findings were highly suggestive of Ewing sarcoma, confirmatory molecular testing (e.g., FISH or RT-PCR for EWSR1 rearrangement) was not performed due to prohibitive out-of-pocket costs and a lack of local laboratory capacity.

Given the evidence of brain metastases, neurology was consulted, and antiepileptic therapy was optimized. She received her first cycle of VAC (Vincristine, Adriamycin, Cyclophosphamide) chemotherapy with Pegfilgrastim support. Radiation Oncology deferred local radiotherapy pending a chemotherapy response assessment. Following chemotherapy, the patient developed febrile neutropenia and was managed with broad-spectrum antibiotics and G-CSF. She also experienced hypoxic respiratory failure secondary to hospital-acquired pneumonia, from which she was gradually weaned. Ophthalmological evaluation confirmed a sixth cranial nerve palsy secondary to intracranial metastatic disease. Throughout her hospitalization, she eventually remained hemodynamically stable with progressive respiratory improvement and was discharged with home oxygen support and close outpatient oncology follow-up.

One month later, she presented to the emergency department with breakthrough seizures and was managed acutely with intravenous benzodiazepines and antiepileptics. The patient’s condition stabilized following treatment; however, despite medical advice for admission and further evaluation, she chose to leave against medical advice.

## Discussion

Our case underscores the aggressive clinical behavior of suspected Ewing sarcoma and the real-world limitations faced in resource-constrained settings. The patient’s extensive bony and intracranial metastases, accompanied by refractory seizures, illustrate both the inherent virulence of this tumor and the systemic barriers that delay optimal intervention.

Conceptually, designating this case as an “unknown primary” presents a diagnostic dilemma. Given the extensive lytic lesions in the vertebrae, sacrum, and right iliac bone, it is highly probable that one of these osseous sites represents the true primary tumor. However, the synchronous presentation of massive, multifocal osseous and extraosseous lesions, combined with the inability to perform a PET-CT scan for comprehensive metabolic staging, prevented the definitive localization of a single origin. We therefore functionally classify this as having “no identifiable primary” to reflect the diagnostic limits encountered in our setting. This aligns with rare reports where no definitive primary tumor site could be localized.^13-17^

Furthermore, what distinguishes our case is the synchronous presentation of widely disseminated disease with dural-based intracranial metastasis. Central nervous system involvement is a highly unusual finding at initial diagnosis, emphasizing the need for comprehensive CNS staging in patients presenting with widespread systemic disease.

The absence of molecular confirmation in our patient highlights a significant gap in low- and middle-income country (LMIC) oncology practice. While the immunophenotype (CD99 diffuse positive, NKX2.2 patchy positive) strongly supports the diagnosis of Ewing sarcoma, gold-standard genetic testing was omitted due to resource barriers.^6,12^ Consequently, the definitive diagnosis remains a small round blue cell tumor highly suspicious for Ewing sarcoma.

The standard of care remains a multimodal approach combining systemic chemotherapy with local control.^1,2,5^ For our patient, the interval-compressed VDC/IE regimen was initiated.^8^ However, challenges faced in LMICs include delayed diagnosis, limited access to advanced imaging, and logistical barriers to delivering complex chemotherapy protocols.

The decision to leave against medical advice further reflects socioeconomic factors that critically disrupt continuity of care in LMIC contexts. The prohibitive out-of-pocket costs associated with prolonged hospitalizations, complex chemotherapy regimens, and the management of severe treatment-related complications often result in catastrophic financial toxicity, compelling patients to abandon necessary medical care.^4,18-23^

There are several limitations to this case report that must be acknowledged. First, as a single observational case study, the findings possess inherent limitations regarding generalizability. Second, the definitive gold-standard molecular confirmation (e.g., FISH or RT-PCR for the EWSR1 rearrangement) was not performed due to severe financial and logistical constraints, meaning the diagnosis relies heavily on clinical, radiological, and immunohistochemical correlation. Third, the inability to procure a PET-CT scan limited our capacity to definitively isolate the primary tumor site. Finally, because the patient chose to leave against medical advice, long-term follow-up and evaluation of ultimate treatment efficacy and overall survival could not be assessed.

Collectively, this case adds to the growing body of reports highlighting diagnostic complexity, unusual metastatic patterns (such as CNS involvement at presentation), and systemic barriers in treating suspected Ewing sarcoma.

## Conclusion

Small round blue cell tumors, such as Ewing sarcoma, remain highly aggressive malignancies with significant diagnostic and therapeutic challenges, especially in low- and middle-income countries. This case highlights the devastating potential of widely metastatic disease with no identifiable primary site at presentation, including rare intracranial involvement. It underscores the need for early recognition, timely molecular confirmation, and integrated multimodal care. Improving awareness, strengthening diagnostic capacity, and addressing socioeconomic barriers are critical steps toward better prognosis and survival for patients facing this formidable disease.

## Data Availability

The original contributions presented in this study are included in the article/supplementary material. Further inquiries can be directed to the corresponding author(s).*
